# Study on the Protective Effect of a New Manganese Superoxide Dismutase on the Microvilli of Rabbit Eyes Exposed to UV Radiation

**DOI:** 10.1155/2015/973197

**Published:** 2015-04-29

**Authors:** Lucia Grumetto, Antonio Del Prete, Giovanni Ortosecco, Francesco Barbato, Salvatore Del Prete, Antonella Borrelli, Antonella Schiattarella, Roberto Mancini, Aldo Mancini

**Affiliations:** ^1^Department of Pharmacy, University of Naples Federico II, Naples, Italy; ^2^Department of Neurosciences and Reproductive and Dentistry Sciences, University of Naples Federico II, Naples, Italy; ^3^Molecular Biology and Viral Oncology Unit, Department of Experimental Oncology, Istituto Nazionale Tumori “Fondazione G. Pascale”, IRCCS, Naples, Italy; ^4^Leadhexa Biotechnologies Inc., QB3@953, 953 Indiana Street, San Francisco, CA 94110, USA

## Abstract

We present a study on the protective effects against UV radiation of a gel formulation containing a new recombinant form of manganese superoxide dismutase on the conjunctiva and corneal epithelia of rabbit eyes. The integrity of the microvilli of both ocular tissues has been considered as an indicator of the health of the tissues. Samples, collected by impression cytology technique, were added of 80 *µ*L of a gel formulation containing superoxide dismutase (2.0 *µ*g/mL) and irradiated with UV rays for 30 minutes and were evaluated with scanning electron microscopy. Wilcoxon test was used to verify the possible occurrence of statistically significant differences between damage for treated and nontreated tissues. Application of gel produces a significant reduction of damage by UV irradiation of ocular epithelia; both epithelia present a significant reduction of damaged microvilli number if treated with the superoxide dismutase gel formulation: the *p* values (differences between damage found for treated and nontreated both ocular tissues) for conjunctiva and cornea samples were *p* ≪ 0.01 and *p* ≪ 0.0001, respectively, at confidence level of 95%. The administration of this gel formulation before UV exposure plays a considerable protective role in ocular tissues of rabbit eye with a significant reduction of the damage.

## 1. Introduction

The biological effects of light and UV radiation (UVR) have been studied worldwide and the potential hazards from both chronic, long-term and single, acute exposure of the eye to them have been the subject of numerous scientific works [1–5]. In rabbit eyes the surface of the cornea was studied under the scanning and transmission electron microscope and compared with the pathological changes which occurred after experimental UVR. After UVR, there are a reduced number of the large impressions or the full-thickness holes of the superficial cells, partly rejected cells, numerous small plasma membrane defects, and the microvilli were destroyed [6]. High levels of mucin are the first and most important line of defense against the oxidative stress of both endogenous and exogenous nature [7]. The second line of defense is represented by enzymes able to preserve the protein structure of mucosal cells in order to save their functionality. The antioxidant ocular enzymes sometimes are not sufficient to counteract the oxidative stress, because the defense systems undergo progressive deterioration resulting in a more and more marked inability to adequately respond to damage by oxidant species, mainly represented by free radicals, determining the peroxidation of the lipids of cell membranes. Corneal endothelium (CE) is a monolayer of cells whose primary function is to maintain corneal transparency by controlling stromal hydration. Oxidative stress is present in the cornea because exposure to light is a significant source for reactive oxygen species (ROS) [8–10]. The superoxide dismutase (SOD) is essential for neutralizing ROS in the cell; it has been demonstrated that the decrease of SOD expression and activity contributes to oxidative stress; of consequence the basal level of apoptotic cells increased and made the cells more susceptible to exogenous UV stress [11].

A new recombinant isoform of human manganese superoxide dismutase (rMnSOD) has been developed [8]. A particular feature of this SOD is its capability to enter the cells thanks to its uncleaved terminal peptide sequence. rMnSOD has been isolated from a human liposarcoma cell line and it has not cytotoxic effects on normal cells [12–14]. Together with its oncotoxic activity, rMnSOD has shown a radioprotective effect on normal cells irradiated by X-rays [12]. It has been proved really effective in scavenging intra- and extracellular superoxide ion (O_2_^−^) and in improving pathological conditions associated with increased oxidative stress [12–16]. SOD converts superoxide ion to H_2_O_2_, which is reduced by mitochondrial glutathione peroxidase into H_2_O. The present study is aimed to investigate if the rMnSOD may be an important support in preventing damage caused by oxidative stress, which in turn can lead to numerous ocular pathologies. The poor therapeutic response exhibited by conventional ophthalmic preparations is due to rapid precorneal elimination, dilution, and nasolacrimal drainage. Consequently, there is a need for frequent instillation of concentrated solutions to achieve the desired therapeutic effect [17, 18]. To overcome these problems, various ophthalmic formulations have been investigated in an attempt to extend the ocular residence time of drugs for topical application to the eye. The use of these dosage forms overcomes the administration problems, but they may cause localized effects, as blurred vision (e.g., ointments), or lack of patient compliance [19]. Therefore in this work we proposed an rMnSOD ophthalmic gel formulation to evaluate the protective effect of the enzyme on microvilli of ocular tissues of rabbit eyes exposed to UVR, based on polyacrylic acid (Carbopol 934) as gelling and viscosity-enhancing agent. Particular attention was paid to the morphological study of microvilli on the apical membrane of the superficial epithelial cells, covered by glycocalyx, that is, the deepest layer of tear film. Indeed, transmembrane mucins are docked at the tips of microvilli and their function is closely related to tear film stability. Tear film binds to the microvilli of epithelium surface, thereby ensuring its wettability [17]. Microvilli, microplicae, and glycocalyx of conjunctival epithelium provide support to tear film preventing defluxion by gravity of the film from cornea [20]. Therefore, our aim has been to demonstrate a possible direct relationship between the treatment with the rMnSOD ophthalmic gel formulation and a possible reduction of epithelial damage, the latter estimated as a reduction of the number of microvilli damaged by UVR, both in conjunctiva and cornea epithelial cells. It is important to note that the microvilli are the membrane structures of epithelia under examination, which are the most sensitive structures to radiation damage. The evaluation has been performed with impression cytology that is a noninvasive, easily reproducible technique, using scanning electron microscopy (SEM) [21, 22].

## 2. Methods

### 2.1. Ophthalmic Gel Preparation

The rMnSOD was obtained as described in the literature [12]. Aqueous solutions of Carbopol 934^©^ 0.1% w/v were prepared by dispersing the required amount of 50.0 mg in 50.0 mL of water for injection (European Pharmacopoeia 7th edition) with continuous stirring (FALC Instruments, Italy) until complete dissolution. The solution was kept under stirring for about 60 hours to allow the polymer hydration. The gel preparation was put through Turboemulsifier Silverston model SL2T and then a solution of NaOH 1.0 M was added dropwise to have a final pH 6.70; the gel was sterilized by UV radiation (gel formulation). An aseptic rMnSOD solution was prepared separately in water for injection and sterilized by Millipore 0.22 *μ*m filters; it was added to the gel formulation to give the required final concentration of 2.0 *μ*g/mL. The blank gel formulation was prepared with the same described procedure, but replacing the protein solution with an equal volume of physiological solution (NaCl 0.9%). Both gel formulations, without and with rMnSOD, were prepared as a single dosage form and in sterile room under laminar flow hood. The developed formulations were primarily evaluated for clarity by visual observation against a black and white background in a well-lit cabinet, drug content by UV spectrophotometry at 280 nm (Shimadzu UV-visible spectrophotometer, Japan), pH (Crison Instruments digital pH meter, Spain).

### 2.2. Experimental Procedure

#### 2.2.1. Preparation of Samples

This study was carried out in slaughterhouse-obtained rabbit eyes. Rabbits, approximately 3 months old and weighing 2.33–3.61 kg, underwent macroscopic examinations, including the presence of conjunctival hyperemia of the eyes, performed at baseline and prior to be sacrificed. Only healthy animals were selected for the study. Thirty-four eyes, immediately enucleated, wrapped in sterile gauze, and sustained by a plastic support, were transported to the laboratory on ice packs. The enucleated eye was looked at under the slit lamp. A fluorescein solution of 0.2% (Fluorofta, SOOFT, Italy) was added to the eyes to evaluate the corneal and conjunctiva surface integrity by slit lamp. The ocular globe was washed with physiological solution (0.9% NaCl). Thirty eyes were treated as follows: the corneal samples were taken by removal of the cornea by using drill Surgistar 7.50 mm without vacuum (Trephine Drill Bits), in order to reduce the trauma of sampling and one specimen of approximately 1 cm^2^ of conjunctiva and one specimen of 1 cm^2^ of cornea were collected.

#### 2.2.2. UVR Treatment

Both cornea and conjunctiva samples, before UV rays, were prepared as follows: they were transferred using the impression cytology technique, [21, 22] by compressing a fragment of cellulose acetate (Millipore^©^) on each specimen and subsequently transferring them by compression on the glass slide for 30 s. Each of the two tissue specimens was further divided into two parts. Half of each specimen was evaluated by SEM to assess if it was suitable for the treatment and does not have any previous damage, it was added of 80 *μ*L gel preparations without rMnSOD, and finally irradiated with UV rays at 11.0 cm of distance (220 V, 30 W, wavelength of 254 nm, for 30 minutes, quartz lamp Jeloprotect, Jelosil, Italy). The other half sample, similarly previously evaluated to be suitable for the experiment, it was added of 80 *μ*L gel preparations containing rMnSOD (2.0 *μ*g/mL) and finally irradiated under the same UV conditions.

#### 2.2.3. SEM Procedure

For SEM the samples were fixed in 3% glutaraldehyde in a 0.065 M (pH 7.4) phosphate buffer for 2 h at room temperature. Slides were washed three times in 0.065 M phosphate buffer (30 min) and placed in 1% OsO_4_ in 0.064 M (pH 7.4) phosphate buffer for 30 min. Samples were dehydrated through a graded series of ethanol and critical-point-dried in a CO_2_ liquid Bemar SPC 1500 apparatus (Bomar Co, Tacome, WA, USA). Specimens were mounted on aluminum stubs with silver-conducting paint, sputtered with a thin (20 nm) gold film, and observed with a Cambridge Mark 250 SEM. Furthermore, four enucleated eyeballs, examined as above described, sustained by a plastic support and wrapped in sterile gauze, were treated as follows: 50 *μ*L gel preparations without rMnSOD were added dropwise to two eyes as control while 50 *μ*L gel preparations containing rMnSOD 2 *μ*g/mL were administered to other two rabbit eyeballs. The formulations were instilled one drop every fifteen minutes to an hour and all four eyeballs were irradiated under the same UV conditions for 30 minutes.

### 2.3. Edema Evaluation by Pachymeter

A pachymeter (Pacline, Optikon 2000 S.p.A, Rome) with a probe at 20 MHz and solid tip were used for determining the amount of corneal thickness, considered as quantitative estimate of eye edema.

**Figure 1:**
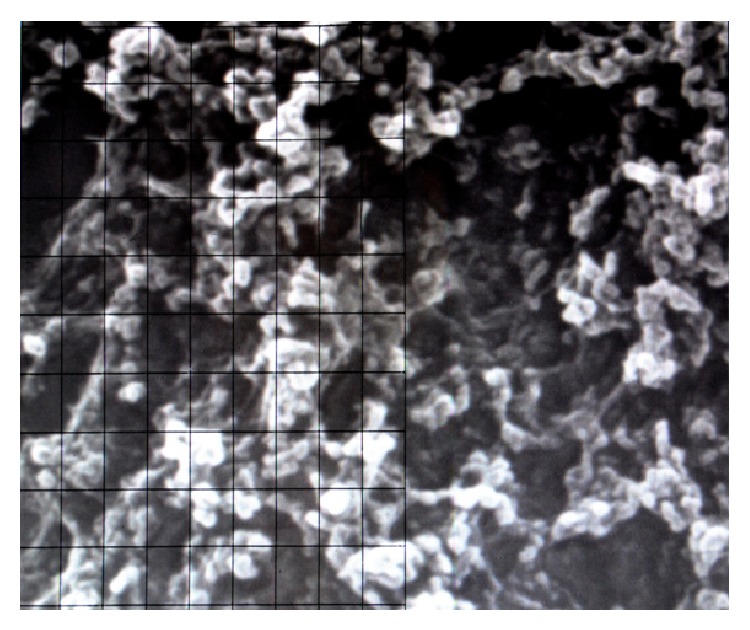
Electron microscope image with the grid for the measurement of the number of conjunctival microvilli of rabbit eye (fields magnification ×7500).

**Figure 2:**
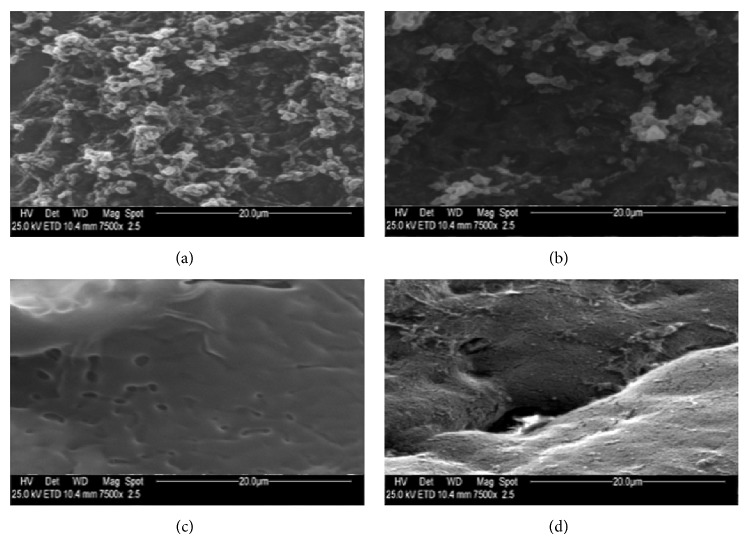
Photography obtained by electron microscopy of microvilli of rabbit eye conjunctival epithelium related to samples of (a) Grade 1 (3000–1500 microvilli); (b) Grade 2 (1500–500 microvilli); (c) Grade 3 (500–100 microvilli); (d) Grade 4 (100–0 microvilli); (magnification ×7500).

**Figure 3:**
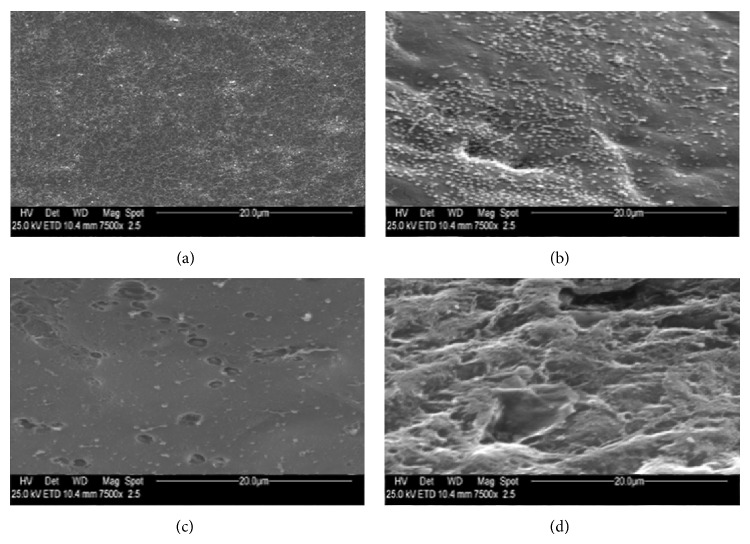
Photography obtained by electron microscopy of microvilli of rabbit eye corneal epithelium related to samples of (a) Grade 1 (3000–1500 microvilli); (b) Grade 2 (1500–500 microvilli); (c) Grade 3 (500–100 microvilli); (d) Grade 4 (100–0 microvilli); (magnification ×7500).

Measurements were made on photographic prints by using a magnifier and a graticule. Microvilli on each sample, after identification at a magnification of ×750 in a 1500 mm^2^ field, were counted, at a magnification of ×7500, in each 230 mm^2^ area of the field selected, as can be seen in Figure 1 for a healthy conjunctival specimen of rabbit eye not exposed to UVR. According to the number of microvilli, samples were classified as Grade 1 (3000–1500 microvilli), Grade 2 (1500–500), Grade 3 (500–100), and Grade 4 (100–0). SEM images of conjunctiva and cornea microvilli at the four grades of UV damage are reported in Figures 2 and 3, respectively.

### 2.4. Statistics

The Wilcoxon test for nominal variables (grades of reduction of microvilli) was performed using the “R” software on Linux Ubuntu 14.04.

## 3. Results

**Figure 4:**
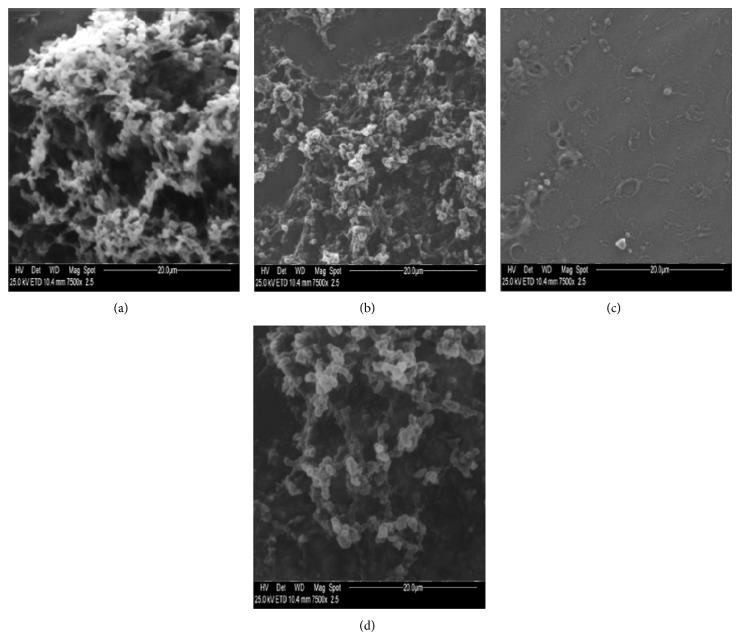
Photography obtained by electron microscopy (magnification ×7500) of microvilli of conjunctiva rabbit eye before (a, b) and after (c, d) UV irradiation. Conjunctival epithelium treated with gel formulation without rMnSOD (a, c) and (b, d) treated with gel formulation containing rMnSOD 2 *μ*g/mL. (a) Evident widespread microvilli. (b) Widespread microvilli on whole surface. (c) Loss of all microvilli of conjunctival surface. (d) Slight decrease of microvilli on conjunctival surface.

**Figure 5:**
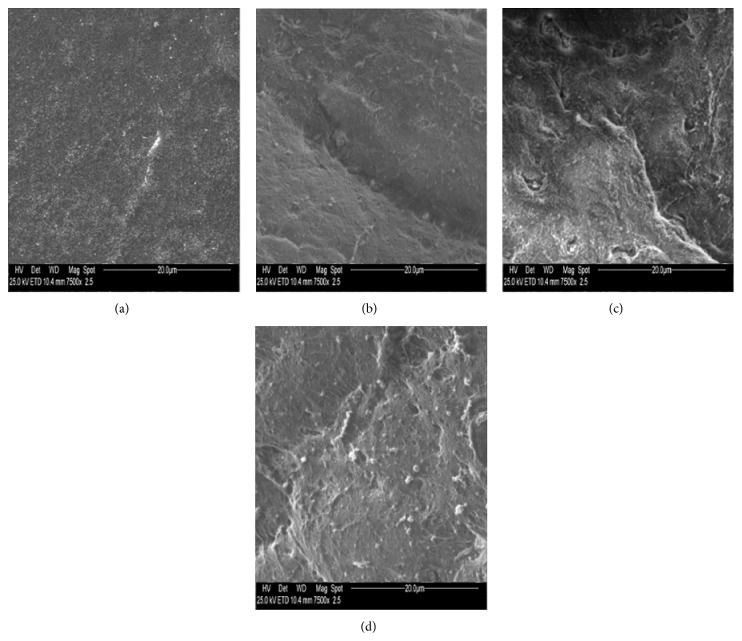
Photography obtained by electron microscopy (magnification ×7500) of microvilli of cornea rabbit eye before (a, b) and after (c, d) UV irradiation. Corneal epithelium treated with gel formulation without rMnSOD (a, c) and with gel formulation containing rMnSOD 2 *μ*g/mL (b, d). (a) A lot of microvilli widespread on whole surface. (b) A groove as intercellular space, microvilli, and microbumps. (c) A cellular split as intercellular space with dryness and numerous wounds, small microbumps, and few microvilli residues. (d) Microvilli and a few small microbumps. There is no appreciable deterioration of epithelium after exposure to UV radiation.

**Figure 6:**
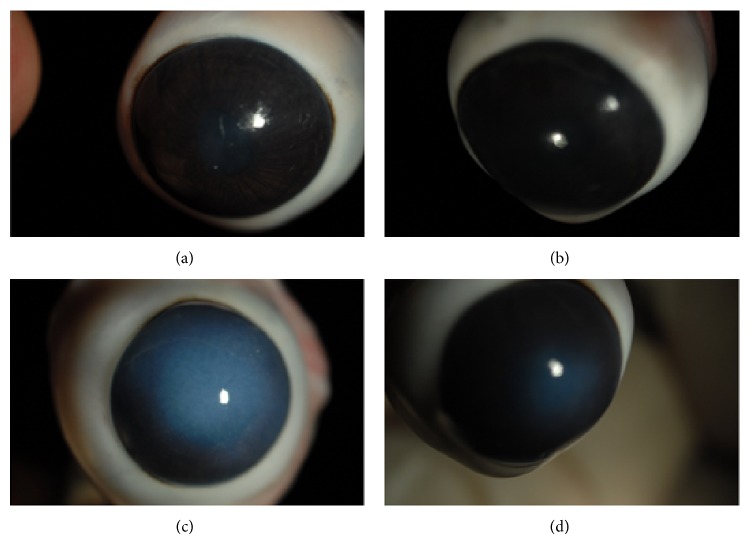
Rabbit eyes before and after exposure to UV radiation. (a) Right eye after administration of gel formulation without rMnSOD and (b) left eye of the same rabbit after administration of gel formulation containing rMnSOD 2 *μ*g/mL before UV irradiation. The same right (c) and left (d) eyes, after exposure to UV rays. (c) Evident corneal suffering with edema. (d) Very mild corneal suffering.

Impression cytology with scanning electron microscopy was found as a highly effective technique to classify and follow up the health of conjunctiva and cornea microvilli and, consequently, to identify and to stage tear film abnormalities. The corneal and conjunctiva specimens, evaluated by SEM, treated with gel formulations without rMnSOD, have been considered as blank, to determine the possible effects of the gel itself on the ocular surface. After UVR exposure, the structure of both conjunctiva and cornea surfaces of all the ocular specimen treated with blank gel formulations showed a noticeable level of damage; in contrast, more than 50% of the samples, the conjunctiva, and cornea structures of samples treated with gel containing 2 *μ*g/mL rMnSOD were completely preserved or with less severe damage, preserving the same morphology of a specimen not exposed to UVR (Figures 4 and 5). These observations suggest that the administration of rMnSOD gel formulation to the ocular tissues before UV exposure plays a considerable protective role on conjunctiva and cornea cells of rabbit eye. For both conjunctiva and cornea samples, the grades of all thirty eyes were reported in Table 1. A Wilcoxon test (analysis of the ranks for nominal variables on an ordinal scale and with paired data) was performed to verify the possible occurrence of statistically significant differences between damage found for treated and nontreated ocular specimens. For each control sample the grade of destruction of microvilli was evaluated, in both conjunctiva and cornea epithelia, before and after UVR exposure; the differences were* D*1 values. The same differences for the samples treated with rMnSOD were* D*2 values. Therefore, we obtained thirty pairs of values to evaluate conjunctiva damage and thirty pairs of values to evaluate cornea damage, each pair referring to the same sample, one of them nontreated and the other one treated with rMnSOD. The differences between damage were evaluated at a confidence level of 95% between the pairs* D*1 −* D*2 referring to the same sample (Δ_0_ =* D*1 −* D*2). The null hypothesis (*H*_0_) formulated in setting phase of the test is the following:* H*_0_: Δ_0_ =* D*1 −* D*2 = 0. The test results were as follows: for the samples of the conjunctiva we reject* H*_0_ with *p* ≪ 0.01 (*p* = 6.451 × 10^−3^), whereas for the cornea samples we reject* H*_0_ with *p* ≪ 0.0001 (*p* = 1.835 × 10^−5^). In particular, for the cornea samples,* D*1 −* D*2 nonzero differences were always with positive sign, indicating that when the microvilli state presented a variation after treatment with rMnSOD, this was always favourable. The difference for paired data of thickness was statistically significant at a confidence level of 95% (*p* ≪ 0.0001). Furthermore, after UVR exposure of the whole eyeball, the structure of cornea surfaces of two eyes treated with blank gel formulations showed a noticeable edema (average thickness of the cornea was 709 ± 37 *μ*m), whereas cornea surfaces of the respective contralateral eye, treated with gel containing 2 *μ*g/mL rMnSOD, showed an edema significantly reduced (average thickness of the cornea was 607 ± 31 *μ*m) as can be seen in Figure 6 (the rabbit central corneal thickness is within the range of published values which ranged from 0.3 to 0.4 mm at its center) [20]. Severity of abnormalities resulted related to the damage of both conjunctiva and cornea epithelial cells, which in turn is related to the number of microvilli [22].

## 4. Discussion

The success of rMnSOD for the prevention of oxidative mechanisms, causing lipid peroxidation, protein oxidation, and DNA damage has been well documented in recent years [12–16]. In order to form a better understanding of a possible protective role of this protein for the treatment of various eye disorders, we investigated the effects of the treatment on corneal and conjunctiva ultrastructure of a gel formulation containing rMnSOD, to prevent the damage caused by UV exposure. Both acute and chronic UV exposure can lead to various ophthalmic abnormalities, associated with photochemical damage to cellular systems with the appearance of typical abnormalities; the type and extent of damage from UV radiant energy are associated with the wavelength, duration, intensity, and size of the exposure [23–25]. The health status of the glycocalyx (microvilli) of the apical membrane of the cornea and conjunctiva mucosa is affected in a negative way from oxidative stress caused by UVR and it is a good indicator of the health of the underlying cells, which, when subjected to long stress, can suffer irreversible damage, causing disease. Furthermore, in Figure 6 the corneal denaturation of untreated eye compared to the only negligible effects of the eye treated with rMnSOD gel formulation is clear, suggesting that the protein may also express a protective activity towards endothelial structures. The results presented in this study provide a scientific evidence demonstrating that the gel formulation containing rMnSOD resulted as a powerful inhibitor of the damage caused by oxidative stress by radiation, both for the conjunctiva and corneal epithelium, manifesting its strongly beneficial effects by significantly reducing the number of missed apical microvilli of both epithelia, showing a less dispersed distribution of microvilli too. As for the pharmaceutical dosage form, it should be underlined that it may play a pivotal role in the efficacy of the treatment. We used Carbopol 934^©^, instead of a simple physiological solution, to increase the viscosity of the vehicle, extending the contact time of the protein with the ocular surface and “trapping” the rMnSOD aqueous solution in the network of polymer. It is important to note that the gel formulation setup was easy to administrate and increase the drug ability to remain on the ocular epithelia. This study demonstrates that gel formulation containing rMnSOD markedly protects ocular tissues from damage caused by oxidative stress of UVR, suggesting that it could be considered as a new therapeutic strategy also in all those ocular diseases in which oxidative stress can occur.

## Figures and Tables

**Table 1:** Grades of destruction of the microvilli of each sample, treated with control gel formulation and with gel formulation containing rMnSOD, of the conjunctiva and the cornea before and after UV irradiation.

	Samples																													
	1	2	3	4	5	6	7	8	9	10	11	12	13	14	15	16	17	18	19	20	21	22	23	24	25	26	27	28	29	30
	Grades																													
Gel formulation without rMnSOD																														
Conjunctiva before UV irradiation	1	2	1	1	1	2	1	1	2	2	2	1	1	1	1	2	2	1	1	2	2	1	1	2	2	1	1	2	2	1
Conjunctiva after UV irradiation	2	3	2	2	3	3	4	3	4	4	3	4	4	3	2	2	3	2	2	3	2	3	4	3	4	2	3	4	4	3
Cornea before UV irradiation	2	2	1	2	2	2	1	1	1	1	1	1	2	2	1	1	2	2	1	1	1	1	1	1	2	1	1	2	2	1
Cornea after UV irradiation	3	2	3	4	3	3	2	2	2	3	3	2	4	4	2	2	3	3	3	4	4	3	2	4	4	3	2	4	4	3
Gel formulation with rMnSOD 2 *µ*g/mL																														
Conjunctiva before UV irradiation	2	1	1	2	1	1	2	1	2	1	1	1	1	1	2	2	1	1	2	2	2	2	2	1	1	1	2	2	1	1
Conjunctiva after UV irradiation	2	3	2	2	2	1	2	2	2	1	2	2	1	2	2	2	1	1	3	2	2	3	4	4	4	3	4	4	3	3
Cornea before UV irradiation	1	2	2	1	1	2	2	2	1	1	2	1	1	2	2	1	1	1	1	1	1	1	1	2	2	1	1	2	2	2
Cornea after UV irradiation	2	2	2	3	2	2	2	2	2	1	2	2	2	3	2	1	1	1	2	1	2	1	2	2	3	1	1	2	2	2
